# A Guide to Urinary Analysis

**Published:** 1873-09

**Authors:** 


### Books Reviewed.

*A Guide to Urinary Analysis, for the use of Physicians and Students.* By Henry G. Piffard, A. M., M. D. New York: Wm. Wood & Co., 1873. Buffalo: H. H. Otis.

The subject of Urinary Analysis receives too little attention from practitioners and students; it being looked upon as requiring too much knowledge and skill in chemical manipulation to be undertaken, and beyond the occasional use of heat and nitric acid in testing for albumen in the urine the majority of physicians may be said never to make an attempt at urinary analysis.

It is the object of the work before us to place in the hands of physicians and students a hand-book which by its simplicity of direction and comparative freedom from error, will make the practical application of its teachings at once easy and pleasant.

The author does not advance his work as one presenting methods whereby the more delicate and accurate analyses are to be made, but as presenting a manual of analysis sufficiently accurate for the purposes of clinical research.

The book is divided into seven chapters with an appendix, embracing various tables. The text treats of the collection and measurement of Urine, Apparatus Reagents and Standard Solutions, Color, Reaction and Specific Gravity, Estimation of Solids, Estimation of Normal Constituents, Detection and Estimation of Abnormal Constituents and Detection of Medicinal and other Substances. The work is one which should be in the library of every practitioner, and can not be too heartily recommended to students in search of a manual of Urinary Analysis.

